# Supplementary material for: Cry3Aa Toxin Is Not Suitable to Control Lepidopteran Pest Spodoptera littoralis (Boisd.)
Source: Plants (Basel). 2022 May 15;11(10):1312. doi: 10.3390/plants11101312 (PMC9145897; doi:10.3390/plants11101312)
Supplement: Supplementary file 1 [file plants-11-01312-s001.zip › Supplementary_materials-Table_S1A-F.pdf]

Supplementary Materials: Cry3Aa Toxin Is Not Suitable to Control Lepidopteran Pests *Spodoptera littoralis* (Boisd.)

Oxana Skoková Habuštová, Zdeňka Svobodová, Dalibor Kodrík and František Sehnal

Table S1: A. Results of Log-rank (Mantel-Cox) test with Bonferroni correction of α level of difference between survival curves of *L. decemlineata* in bioassay 1 with recombinant (Bonferroni correction: α = 0.0024) and natural Cry3Aa (Bonferroni correction: α = 0.0011) in artificial diet and with Cry3Aa expressed in GM potato SNL. Statistically significant p-values are highlighted in bold.

Toxin	Compared treatments	Chi square (χ^2)	df	p
Recombinant	Control x 1 µg/g	10.51	1	**0.0012**
Cry3Aa	Control x 2 µg/g	28.01	1	**<0.0001**
	Control x 4 µg/g	40.69	1	**<0.0001**
	Control x 6 µg/g	59.99	1	**<0.0001**
	Control x 8 µg/g	59.95	1	**<0.0001**
	Control x 10 µg/g	69.29	1	**<0.0001**
	1 µg/g x 2 µg/g	7.58	1	0.0059
	1 µg/g x 4 µg/g	17.14	1	**<0.0001**
	1 µg/g x 6 µg/g	33.15	1	**<0.0001**
	1 µg/g x 8 µg/g	23.01	1	**<0.0001**
	1 µg/g x 10 µg/g	41.84	1	**<0.0001**
	2 µg/g x 4 µg/g	1.89	1	0.1697
	2 µg/g x 6 µg/g	9.33	1	**0.0023**
	2 µg/g x 8 µg/g	3.66	1	0.0556
	2 µg/g x 10 µg/g	14.86	1	**<0.0001**
	4 µg/g x 6 µg/g	3.01	1	0.0827
	4 µg/g x 8 µg/g	0.15	1	0.6946
	4 µg/g x 10 µg/g	6.82	1	0.0090
	6 µg/g x 8 µg/g	2.27	1	0.1322
	6 µg/g x 10 µg/g	1.41	1	0.2346
	8 µg/g x 10 µg/g	6.88	1	0.0087
Natural	Control x 0.01 µg/g	5.89	1	0.0152
Cry3Aa	Control x 0.1 µg/g	22.53	1	**<0.0001**
	Control x 0.5 µg/g	38.69	1	**<0.0001**
	Control x 1 µg/g	97.75	1	**<0.0001**
	Control x 2 µg/g	69.50	1	**<0.0001**
	Control x 4 µg/g	88.00	1	**<0.0001**
	Control x 6 µg/g	77.73	1	**<0.0001**
	Control x 8 µg/g	80.72	1	**<0.0001**
	Control x 10 µg/g	81.21	1	**<0.0001**
	0.01 µg/g x 0.1 µg/g	6.46	1	0.0110
	0.01 µg/g x 0.5 µg/g	14.26	1	**0.0002**
	0.01 µg/g x 1 µg/g	69.39	1	**<0.0001**
	0.01 µg/g x 2 µg/g	40.99	1	**<0.0001**
	0.01 µg/g x 4 µg/g	60.08	1	**<0.0001**
	0.01 µg/g x 6 µg/g	55.63	1	**<0.0001**

0.01 µg/g x 8 µg/g	51.58	1	**<0.0001**
0.01 µg/g x 10 µg/g	58.2	1	**<0.0001**
0.1 µg/g x 0.5 µg/g	4.17	1	0.0412
0.1 µg/g x 1 µg/g	82.56	1	**<0.0001**
0.1 µg/g x 2 µg/g	36.55	1	**<0.0001**
0.1 µg/g x 4 µg/g	68.35	1	**<0.0001**
0.1 µg/g x 6 µg/g	63.06	1	**<0.0001**
0.1 µg/g x 8 µg/g	53.67	1	**<0.0001**
0.1 µg/g x 10 µg/g	70.92	1	**<0.0001**
0.5 µg/g x 1 µg/g	29.26	1	**<0.0001**
0.5 µg/g x 2 µg/g	7.23	1	0.0072
0.5 µg/g x 4 µg/g	22.97	1	**<0.0001**
0.5 µg/g x 6 µg/g	23.67	1	**<0.0001**
0.5 µg/g x 8 µg/g	15.30	1	**<0.0001**
0.5 µg/g x 10 µg/g	27.64	1	**<0.0001**
1 µg/g x 2 µg/g	13.68	1	**0.0002**
1 µg/g x 4 µg/g	0.19	1	0.6606
1 µg/g x 6 µg/g	0.56	1	0.4533
1 µg/g x 8 µg/g	1.15	1	0.2836
1 µg/g x 10 µg/g	0.72	1	0.3955
2 µg/g x 4 µg/g	9.24	1	0.0024
2 µg/g x 6 µg/g	13.34	1	**0.0003**
2 µg/g x 8 µg/g	4.38	1	0.0363
2 µg/g x 10 µg/g	14.55	1	**0.0001**
4 µg/g x 6 µg/g	1.21	1	0.2707
4 µg/g x 8 µg/g	0.42	1	0.5186
4 µg/g x 10 µg/g	1.29	1	0.2554
6 µg/g x 8 µg/g	1.55	1	0.2132
6 µg/g x 10 µg/g	0.09	1	0.7705
8 µg/g x 10 µg/g	2.12	1	0.1455

Table S1: B. Result of statistical tests of three *S. littoralis* populations in bioassay 2 with 8 µg/g recombinant and natural Cry3Aa in artificial diet. Statistically significant p-values are highlighted in bold (Bonferroni correction: α = 0.017 in Chi-square test and Chi-square test for trend). The type of test used for each parameter and abbreviations of *S. littoralis* populations are described in section 5. Material and Methods.

Population	Examined parameter	Test statistics	df	p	Post-hoc test (p) [1]
NRC	Initial larval weight (mg)	F = 1.24	2,113	0.293	
	Body weight gain (mg)[2]				control x recombinant Cry3Aa: χ^2 = 0.18, df = 1, p = 0.668<br>control x natural Cry3Aa: χ^2 =0.39, df = 1, p = 0.532<br>recombinant Cry3Aa x natural Cry3Aa: χ^2 = 1.03, df = 1, p = 0.311
	ECI (%)[3]	F = 3.49	2,11	0.067	
	Weight increment (mg)	F = 9.82	2,113	**< 10^{-3}**	recombinant Cry3Aa x control: **< 10^{-3}**<br>recombinant Cry3Aa x natural Cry3Aa: **< 10^{-3}**
	Maximal body weight (mg)	F = 10.07	2,113	**< 10^{-3}**	recombinant Cry3Aa x control: **< 10^{-3}**<br>recombinant Cry3Aa x natural Cry3Aa: **< 10^{-3}**
	Length of fifth instar (days)	F = 0.51	2,113	0.604	
	Length of sixth instar (days)	F = 0.124	2,113	0.883	
	Length of prepupal stage (days)	F = 1.87	2,113	0.159	
	Larval mortality (%)	χ^2 = 6.15	2	**0.046**	n. a.[4]
	Pupal weight (mg)	F = 3.45	2,113	**0.035**	recombinant Cry3Aa x control: **0.017**<br>recombinant Cry3Aa x natural Cry3Aa: **0.047**
	Length of pupal stage (days)	F = 209.25	2,110	**< 10^{-3}**	recombinant Cry3Aa x control: **< 10^{-3}**<br>recombinant Cry3Aa x natural Cry3Aa: **< 10^{-3}**<br>control x natural Cry3Aa: **0.045**
	Pupal mortality (%)	χ^2 = 6.66	2	**0.036**	n. a.
	No. of laid eggs per female per day	F = 0.11	2,46	0.896	
	No. of hatched eggs per female per day	F = 0.04	2,46	0.956	
SE	Initial larval weight (mg)	F = 1.87	2,87	0.160	
	Body weight gain (mg)				control x recombinant Cry3Aa: χ^2 = 8.08, df = 1, p = **0.005**

					control x natural Cry3Aa:<br>χ^2 =9.13, df = 1, p = **0.003**<br>recombinant Cry3Aa x natural Cry3Aa:<br>χ^2 = 30.18, df = 1, p **< 10^{-3}**
	ECI (%)	F = 1.84	2,11	0.205	
	Weight increment (mg)	F = 6.96	2,87	**0.002**	recombinant Cry3Aa x natural Cry3Aa: **0.029**<br>control x natural Cry3Aa: **0.002**
	Maximal body weight (mg)	F = 7.14	2,87	**0.001**	recombinant Cry3Aa x natural Cry3Aa: **0.025**<br>control x natural Cry3Aa: **0.002**
	Length of fifth instar (days)	F = 7.21	2,87	**0.001**	control x recombinant Cry3Aa: **0.003**<br>natural Cry3Aa x recombinant Cry3Aa: **0.003**
	Length of sixth instar (days)	F = 1.65	2,87	0.200	
	Length of prepupal stage (days)	F = 1.04	2,82	0.359	
	Larval mortality (%)	χ^2 = 2.05	2	0.358	
	Pupal weight (mg)	F = 4.65	2,82	**0.012**	control x natural Cry3Aa: **0.035**
	Length of pupal stage (days)	F = 2.00	2,79	0.143	
	Pupal mortality (%)	χ^2 = 1.70	2	0.426	
	No. of laid eggs per female per day	F = 0.75	2,26	0.480	
	No. of hatched eggs per female per day	F = 0.61	2,26	0.551	
SF	Initial larval weight (mg)	F = 0.59	2,93	0.557	
	Body weight gain (mg)				control x recombinant Cry3Aa:<br>χ^2 = 0.74, df = 1, p = 0.39<br>control x natural Cry3Aa:<br>χ^2 =0.41, df = 1, p = 0.522<br>recombinant Cry3Aa x natural Cry3Aa:<br>χ^2 = 0.05, df = 1, p = 0.821
	ECI (%)	F = 0.49	2,12	0.623	
	Weight increase (mg)	F = 0.63	2,93	0.534	
	Maximal body weight (mg)	F =0.64	2,93	0.532	
	Length of fifth instar (days)	F = 1.38	2,93	0.256	
	Length of sixth instar (days)	F = 0.10	2,93	0.903	
	Length of prepupal stage (days)	F = 1.43	2,93	0.243	
	Larval mortality (%)	χ^2 = 1.40	2	0.500	

Pupal weight (mg)	F = 0.60	2,83	0.551	
Length of pupal stage (days)	F = 3.99	2,93	**0.022**	natural Cry3Aa x recombinant: **0.033**
Pupal mortality (%)	χ^2 = 2.49	2	0.287	
No. of laid eggs per female per day	F = 1.64	2,55	0.202	
No. of hatched eggs per female per day	F = 1.33	2,55	0.274	

[1]Treatment with significantly higher value (mg, days) is stated as the first one. Results of post-hoc tests are given only for significantly different treatments; [2]Body weight gain: overall Chi-square test for trend of all three treatments is not possible; [3]ECI: efficiency of food conversion to biomass, [4]n. a.: not available: all post-hoc tests were not significant. N - population of *S. littoralis* reared in our laboratory; NRC - population of *S. littoralis* obtained from National Research Centre, Egypt; CU-population of *S. littoralis* obtained from Cairo University, Egypt; SE - sensitive population of *S. littoralis* obtained from Egypt; SF - sensitive population of *S. littoralis* obtained from France.

Table S1: C. Result of statistical tests of five *S. littoralis* populations in bioassay 3 with Cry3Aa expressed in GM potato SNL. Statistically significant p-values are highlighted in bold. The type of test used for each parameter and abbreviations of *S. littoralis* populations are described in section 5. Material and Methods.

Population	Examined parameter	Test statistics	df	p
N	Initial larval weight (mg)	F = 1.61	1,34	0.213
	Body weight gain (mg)	χ^2 = 0.73	1	0.392
	ECI (%)[1]	F = 0.04	1,8	0.852
	Weight increment (mg)	F = 0.16	1,34	0.692
	Maximal body weight (mg)	F = 0.14	1,34	0.711
	Length of fifth instar (days)	F =0.52	1,34	0.476
	Length of sixth instar (days)	F < 10^{-3}	1,34	0.992
	Length of prepupal stage (days)	F = 2.27	1,34	0.141
	Larval mortality (%)	χ^2 = 0.63	1	0.426
	Pupal weight (mg)	F = 0.46	1,34	0.503
	Length of pupal stage (days)	F = 0.49	1,34	0.490
	Pupal mortality (%)	n. a.[2]		
	No. of laid eggs per female per day	F = 0.47	1,13	0.504
	No. of hatched eggs per female per day	F = 0.01	1,13	0.926
NRC	Initial larval weight (mg)	F = 1.57	1,30	0.220
	Body weight gain (mg)	χ^2 = 11.96	1	**< 10^{-3}**
	ECI (%)	F = 5.44	1,2	0.156
	Weight increase (mg)	F = 1.06	1,30	0.311
	Maximal body weight (mg)	F = 1.02	1,30	0.322
	Length of fifth instar (days)	n. a.		
	Length of sixth instar (days)	F = 5.17	1,30	**0.031**
	Length of prepupal stage (days)	F = 1.60	1,30	0.216
	Larval mortality (%)	χ^2 = 1.68	1	0.194
	Pupal weight (mg)	F = 0.94	1,30	0.339
	Length of pupal stage (days)	F = 39.32	1,30	**< 10^{-3}**
	Pupal mortality (%)	n. a.		
	No. of laid eggs per female per day	F = 1.47	1,10	0.253
	No. of hatched eggs per female per day	F = 0.17	1,10	0.690
CU	Initial larval weight (mg)	F = 1.25	1,31	0.271
	Body weight gain (mg)	χ^2 = 1.20	1	0.273
	ECI (%)	F = 0.37	1,6	0.563
	Weight increase (mg)	F = 0.51	1,31	0.480
	Maximal body weight (mg)	F = 0.49	1,31	0.491
	Length of fifth instar (days)	F = 0.43	1,31	0.515
	Length of sixth instar (days)	F < 10^{-2}	1,31	0.952
	Length of prepupal stage (days)	F = 1.87	1,31	0.182
	Larval mortality (%)	χ^2 = 0.27	1	0.602
	Pupal weight (mg)	F < 10^{-2}	1,31	0.946
	Length of pupal stage (days)	F = 3.95	1,31	0.056
	Pupal mortality (%)	n. a.		
	No. of laid eggs per female per day	F = 0.99	1,13	0.340
	No. of hatched eggs per female per day	F = 4.39	1,13	0.056
SE	Initial larval weight (mg)	F = 0.31	1,50	0.582
	Body weight gain (mg)	χ^2 = 11.94	1	**< 10^{-3}**
	ECI (%)	F = 0.28	1,8	0.612

	Weight increase (mg)	F = 0.99	1,50	0.325
	Maximal body weight (mg)	F = 1.16	1,50	0.286
	Length of fifth instar (days)	F = 0.04	1,50	0.847
	Length of sixth instar (days)	F = 0.01	1,50	0.933
	Length of prepupal stage (days)	F = 1.81	1,50	0.185
	Larval mortality (%)	$\chi^2 = 1.46$	1	0.228
	Pupal weight (mg)	F = 0.41	1,50	0.527
	Length of pupal stage (days)	F = 13.60	1,49	**0.001**
	Pupal mortality (%)	$\chi^2 = 0.49$	1	0.487
	No. of laid eggs per female per day	F = 0.01	1,23	0.910
	No. of hatched eggs per female per day	F = 1.02	1,23	0.322
SF	Initial larval weight (mg)	F = 1.18	1,54	0.283
	Body weight gain (mg)	$\chi^2 = 9.43$	1	**0.002**
	ECI (%)	F = 0.78	1,6	0.410
	Weight increase (mg)	F = 3.63	1,54	0.062
	Maximal body weight (mg)	F = 3.68	1,54	0.060
	Length of fifth instar (days)	F =5.02	1,54	**0.029**
	Length of sixth instar (days)	F = 2.84	1,54	0.098
	Length of prepupal stage (days)	F = 53.14	1,53	**$< 10^{-3}$**
	Larval mortality (%)	$\chi^2 = 0.34$	1	0.554
	Pupal weight (mg)	F = 4.00	1,53	0.051
	Length of pupal stage (days)	F = 6.27	1,31	**0.018**
	Pupal mortality (%)	$\chi^2 = 6.45$	1	**0.011**
	No. of laid eggs per female per day	F = 0.82	1,15	0.379
	No. of hatched eggs per female per day	F = 0.24	1,15	0.63

[1]ECI: efficiency of food conversion to biomass; [2]n. a.: not available: no variability in data

N - population of *S. littoralis* reared in our laboratory; NRC - population of *S. littoralis* obtained from National Research Centre, Egypt; CU- population of *S. littoralis* obtained from Cairo University, Egypt; SE - sensitive population of *S. littoralis* obtained from Egypt; SF - sensitive population of *S. littoralis* obtained from France.

S1: D. Preparation of semi-artificial diet for *L. decemlineata* larvae (description).

Batches of 150 ml were used per a standard test of each toxin. A solution of 1.98 g agar in 70.0 ml deionized water was prepared in an autoclaved bottle in a microwave oven, cooled by adding 40.0 ml cold water, and placed on a water bath of 48–56°C. Dry diet components (21.09 g) were added under continual mixing. The bottle was closed and shaken in the water bath to achieve homogenous suspension. Formaldehyde (0.38 ml; 37 %) and potassium hydroxide (0.68 ml; 18.3 %) were added, the bottle was closed, the contents were mixed thoroughly, and divided into aliquots of 13.0 g and kept on water bath at 50–53°C. Diets containing different amounts of Cry3Aa were prepared by adding specific amounts of the Cry3Aa solution.to achieve the desired concentration. The content was mixed thoroughly with a spoon and dispensed in 0.3 ml portions into wells of a titer plate (#K082248.1 from P-lab, 48 wells, well volume 1.76 ml, area 0.98 cm^2). Eppendorf pipette and 1 ml tips with cut ends were used for pipetting. Plates with diet were kept in the refrigerator for not more than 2 days before use.

S1: E. Preparation of a semi-artificial agar bean diet for *S. littoralis* larvae (description).

500 g of small white beans cultivar Aidagold (La Food s.r.o. Česká Republika) were cooked until soft. The excess water was removed, and the beans were homogenized in a blender. The 75 g dry yeast was cooked in 300 ml distilled water and thoroughly mixed with the bean base. The soluble ingredients, namely 7.5 g ascorbic acid, 1.25 g sorbic acid and 4.75 g methylparaben were successively added into the bean base. Hot solution of 30 g agar dissolved in 500 ml water was added and the hot diet was poured into three Petri dishes (20 cm diameter). After cooling at room temperature, the dishes were stored in a refrigerator (4 °C) for up to one month.

S1: F. Preparation of a semi-artificial soy powder and corn bran diet for *S. littoralis* larvae (description).

50 g of agar was prepared by boiling in 1.6 l water under continuous mixing by beater for at least 5 minutes. Then it was cooled down in water bath to room temperature in sink under frequent mixing to avoid solidification of the agar on pot walls. The 840 g of dry premix (665 g soya cake, 665 g pinole, 333 g wheat germs, 250 g brewer's yeast, 26 g benzoic acid, 11.4 g nipagin, 46.8 g ascorbic acid) was poured into a blender pot together with 1.6 l of agar and homogenized. Other dry components, namely 30 g casein, 12 g Wesson salt mix, 30 g vitamin premix (833.5 g D-Glucose, 45g ascorbic acid, 5 g benzoic acid, 4.45 g nicotinic acid, 5 g inositol, 3 g DL-pantothenic acid, 2.25 g menadione, 1 g riboflavine, 1 g pyridoxine, 1 g thiamine hydrochloride, 1.8 g vitamin A acetate, 0.125 g calciferol, 0.02 g D-biotine, 0.09 g folic acid, 0.00135 g vitamin B12, 75 g choline chloride, 22 g DL α tocopherol acetate), and antibiotics (0.3 g Ampiphac and 0.4 g Acti Tetra®) were added slowly. Liquid components – 8 ml linseed oil and 1.5 ml formaldehyde were added. Artificial diet was poured into three Petri dishes (20 cm diameter). The dishes were stored in a refrigerator (4 °C) for up to one month.
